# This is how breast cancer should not be managed!

**DOI:** 10.11604/pamj.2020.37.261.26045

**Published:** 2020-11-23

**Authors:** Sherif Monib, Hany Habashy

**Affiliations:** 1 St Albans City Hospital, West Hertfordshire Hospitals NHS Trust, St Albans, United Kingdom,; 2 General Surgery Department, Elfayoum University Hospital, Fayoum, Egypt

**Keywords:** Breast cancer, management, surgery

## Image in medicine

We are presenting a case of a 54-year-old lady who was referred to us following a recent catastrophic incomplete excision done by a general surgeon for a locally advanced right breast 62mm invasive ductal carcinoma, grade III, estrogen and progesterone positive, Her2 negative disease, with no axillary procedure initially carried out. We treated her with completion mastectomy and axillary lymph node clearance followed by adjuvant chemotherapy and radiotherapy as well as hormonal treatment. Locally advanced breast cancer is a complex entity which needs expert management; appropriate imaging at presentation, including digital mammogram as well as breast ultra sound scan, followed by breast and axillary biopsies to delineate cancer characteristics is standard practice. Staging computed tomography (CT) chest, abdomen and pelvis scan is reserved for locally advanced disease or nodal involvement. Multi-disciplinary team input discussing options of neo-adjuvant (endocrine/chemotherapy) treatment to downstage the tumour before surgical treatment ensures a better outcome. Also, breast to tumour volume ratio assessment as well as the use of oncoplastic breast resection techniques can avoid such a drastic outcome. Silk sutures are not used any more to close breast incisions; also if a drain is needed, a small-bore closed system suction drain should be placed rather than a corrugated rubber drain.

**Figure 1:**
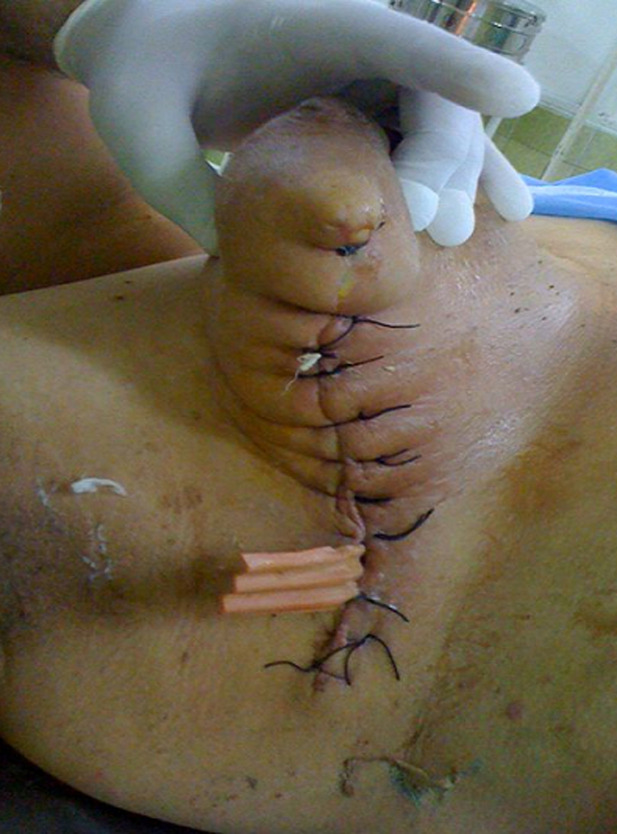
postoperative picture following right breast wide local excision

